# Supplementary material for: Effect of tetanic stimulation prior to train-of-four monitoring on the time to muscle response stabilization with neuromuscular blockade with rocuronium in patients aged 60 to 80 years: A prospective randomized controlled trial
Source: PLoS One. 2024 Jun 14;19(6):e0303161. doi: 10.1371/journal.pone.0303161 (PMC11178177; doi:10.1371/journal.pone.0303161)
Supplement: S1 Dataset — (DOCX) [file pone.0303161.s001.docx]

**Dataset about effect of tetanic stimulation prior to Train-of-Four sequence on stabilization time of muscle response in patients aged 60-80**

Angela Henrique Silva Ribeiro^1*^, Paulo Alípio Germano Filho^1^, Nubia Verçosa Figueiredo^2^, Angelo Jorge Queiroz Rangel Micuci^1^, Ismar Lima Cavalcanti^1^

^1^ Universidade Federal Fluminense, Anesthesiology Dept., Niterói-RJ, Brazil.

^2^ Universidade Federal do Rio de Janeiro, Anesthesiology Dept., Rio de Janeiro-RJ, Brazil.

**Corresponding author**

Angela Henrique Silva Ribeiro

ORCID: https://orcid.org/0000-0003-4253-8792

MSc, MD anesthesiologist

Mariz e Barros street, 775

angelahenriqueanest@gmail.com

+55021 998400223

**Keywords**

Neuromuscular Blocking Agents; Neuromuscular Blockade; General Anesthesia; Neuromuscular Monitoring; Rocuronium;

**Abstract**

The most recent guideline for good clinical practice for research on new neuromuscular blocking drugs, published in 2007, recommends in neuromuscular monitoring that the muscle response be stabilized (T1 height) using a 50Hz tetanic stimulus before calibration and injection of the neuromuscular blocking agent. Therefore, it was executed a randomized, single blind controlled clinical trial in order to evaluate the interference of tetanic stimulation in patients aged 60 to 80 years in the stabilization time of T1 height, onset of action of neuromuscular blocking agent and recovery from neuromuscular blockade after a single dose of rocuronium. The setting was a multicenter study, in tertiary public hospitals, at Rio de Janeiro-RJ (Brazil), from December 2019 to February 2022, with sixty patients aged 60 to 80 years undergoing elective surgeries with general anesthesia, in which they were randomly divided into two groups, one to receive the tetanic stimulus intervention (group T) and one not (group C), before calibration of the neuromuscular monitor and injection of the neuromuscular blocking agent. The data obtained represents the outcome measures: Time to obtain T1 height stabilization, time to onset of action of the neuromuscular blocking agent, recovery times from neuromuscular blockade with or without the interference of the tetanic stimulus.

**Specifications table**

*Every section of this table is mandatory. Please enter information in the right-hand column and remove all the instructions in blue, italicized text.*

| **Subject** | Anesthesiology and Pain Medicine |
| --- | --- |
| **Specific subject area** | Neuromuscular Blocking Agents are used in anesthesia to facilitate endotracheal intubation, and maintenance of controlled mechanical ventilation in intensive care and monitoring is needed. |
| **Type of data** | Table |
| **How the data were acquired** | It was performed a prospective, randomized, single-blind, multicenter, clinical trial, approved by ethics committees and registered at REBEC, with sixty patients submitted to elective surgery under general anesthesia, with routine preoperative evaluation. Patients were randomly assigned using software *GraphPad Prism QuickCalcs*, into one of two groups: the first to receive tetanic stimulus and the other not to stabilize the signal at TOF-Watch Sx monitor. Patients were submitted to anesthesia protocol in the operating room by a single anesthesiologist, and then neuromuscular monitoring was performed by acceleromyography with TOF-Watch monitor, using the TOF sequence stimulation pattern. |
| **Data format** | Raw |
| **Description of data collection** | A personal computer, by means of an interface, recorded the data, from the TOF-Watch SX® monitor, of the effect of electrical stimulation of 2 Hz for 1.5 s repeated every 15 s through TOF stimulation: T1, T2, T3, T4. After general anesthesia and hypnosis assured with bispectrals index below 60, TOF stimulation of the ulnar nerve was initiated for 1 minute. In the control group, calibration was triggered to obtain supramaximal stimulation 100% of T1, and signal stabilization was waited when the maximum variation of 5% in T1 was maintained for at least 2 minutes. In the intervention group, a tetanic stimulus of 50Hz for 5s was applied and then TOF sequence with a supramaximal stimulation with 200ms duration, 2Hz frequency and 15s interval between them was initiated. |
| **Data source location** | · Institution: Hospital Universitário Federal Gaffrée e Guinle (HUGG-UNIRIO); Hospital Universitário Pedro Ernesto (HUPE-UERJ)  · City/Town/Region: Rio de Janeiro-RJ  · Country: Brazil |
| **Data accessibility** | Repository name: Mendeley Data  Data identification number: DOI: 10.17632/fyhpfpk5cm.1  Direct URL to data: https://data.mendeley.com/datasets/fyhpfpk5cm |
| **Related research article** | A.H.S Ribeiro, N.V. Figueiredo, P.A. Germano Filho,A.J.Q.R. Micuci, I.L. Cavalcanti, Effect of tetanic stimulation prior to Train-of-Four sequence on stabilization time of muscle response in patients aged 60-80. In Press |

**Value of the data**

*Provide up to 6 bullet points explaining why these data are of value to the scientific community. At a minimum you must answer the first 3 bullet point questions below (then those questions should be deleted). Please keep your points brief – ideally, each one should be no longer than 400 characters. Avoid any conclusions or inferences.*

*· Why are these data useful?- To improve research in neuromuscular monitoring*

*· Who can benefit from these data? – Development of new neuromuscular blockers*

*· How can these data be used/reused for further insights and/or development of experiments?*

*We seek to help avoiding mistakes and misunderstandings in monitoring neuromuscular blockade, improve the research of new neuromuscular blockers and keep good quality in neuromuscular monitoring.*

**Objective**

Neuromuscular blocking agents (NBA) are widely used in health care settings around the world for general anesthesia, facilitation of endotracheal intubation, and maintenance of controlled mechanical ventilation in intensive care [1,2].

Neuromuscular blockage monitoring is essential for safe surgery protocol, reliable to ensure its degree and reversibility, avoiding complications and excessive hospitals costs. Train-of-four (TOF) sequence is the most commonly stimulation pattern in clinical practice, which assesses the degree of muscle strength by the ratio of the last muscle contraction over the first contraction (T4/T1) [3-10].

The 2007 guideline for good practice regarding pharmacodynamic studies [11] recommends calibration and stabilization of the muscle response to electrical stimulation, before injection of the NBA. However, it may take up to 20 minutes to occur, so applying 5hz tetanic stimulus can decrease this period [12].

Pharmacokinetics and pharmacodynamics of NBAs are altered with aging and difficult to perform researches due to comorbidities [13]. Whereas studies with elderly groups using tetanic stimulation have not been discovered up to the present moment, the primary objective was to determine the time to stabilize the muscle response using tetanic stimulation prior to TOF monitoring in patients over 60 years of age. As secondary objectives, to determine the onset of action of NBAs, clinical duration, pharmacological duration, initial and final T1 height, and its reversibility in those patients.

**Data description**

The datasheet “*Dataset.xlsx*” contains the data collected for this clinical trial. Each column represents a category of the data collected and each line represents an individual participant and their data collected throughout the columns. Above are descriptions for each category of data in columns collected for this clinical trial:

- N: each patient that participated in this research;
- Group: represents the category of groups in which the patients were allocated during the research: Control (C) or Intervention (I);
- Age: the length of time during which each one of patients has existed, measured by years from birth;
- BMI: body mass index;
- Sex: a male (M) or female (F) label assigned to the patient at birth;
- ASA: American Society of Anesthesiology physical status 1 to 3;
- Creatinine Clearance: volume of blood plasma cleared of creatinine per unit time;
- Rocuronium Dosage: the amount of Rocuronium given to each patient, which is a non-depolarizing neuromuscular blocker widely used to produce muscle relaxation to help facilitate surgery and ventilation of the lungs in elective and emergent situations;
- T Stabilization: the period of time it took to stabilize the muscle electrical signal;
- T of inj to TOF 0: time of injection of neuromuscular blocking agent util it reaches a TOF (train-of-four) 0;
- Rocuronium Duration (95%): duration of neuromuscular blocking agent rocuronium
- 2 responses to TOF: represented by two bars in the monitorization;
- Clinical Duration (25%): duration of clinical rocuronium action ( T1 25% of recovery-neuromuscular function);
- Time 75% T1: time to reach the level of 75% of first stimulus in TOF (T1)
- T TOF 40%: time to reach a TOF of 40%;
- T TOF 50%: time to reach a TOF of 50%;
- T TOF 60%: time to reach a TOF of 60%;
- T TOF 70%: time to reach a TOF of 70%;
- T TOF 80%: time to reach a TOF of 80%;
- T TOF 90%: time to reach a TOF of 90%;
- T TOF 100%: time to reach a TOF of 100%;
- Initial T1: level of initial T1;
- Final T1: level of final T1;

This description is also found in the document “*Data Description.docx*”, and the steps to reproduce in the document “*TOF Methods Description.docx*”.

**Experimental design, materials and methods**

A prospective, randomized, single-blind, multicenter, clinical trial was performed, approved by the ethics committees of Hospital Universitário Federal Gaffrée e Guinle (CAAE 03260918.4.0000.5253) and Hospital Universitário Pedro Ernesto (CAAE 03260918.4.3001.5259), Rio de Janeiro-RJ, Brazil, and registered at clinical trials Brazilian platform REBEC (RBR-35msdt).

Sixty patients submitted to elective surgery under general anesthesia, with routine preoperative evaluation, were consecutively recruited and informed consents obtained. Randomization into two groups was performed using the program *GraphPad Prism QuickCalcs* (GraphPad software, Inc. La Jolla, California, USA). Patients were then allocated into one of two groups: one that received tetanic stimulus (n=30) or the other that did not receive tetanic stimulus (n=30). A single researcher conducted the data collection and was aware of the group randomly assigned.

Complementary exams prior to surgery were: Blood cell count, coagulogram, urea, creatinine clearance, sodium, potassium, glucose, chest teleradiograph, and electrocardiogram.

This study followed the Stockholm protocol guideline [11].

Inclusion & Exclusion Criteria

Inclusion criteria were patients between 60 to 80 years old, all genders, ASA (American Society of Anesthesiology) physical status I to III, BMI (Body Mass Index) between 18.5 to 29, undergoing total venous general anesthesia with an presumed surgical duration over 1h.

Exclusion criteria were presence of neuromuscular diseases, renal or hepatic dysfunction, use of furosemide, aminoglycosides, aminophylline, azathioprine, cyclophosphamide, anti-inflammatory drugs and magnesium, allergy to the drugs used in the study, and history or predictors of difficult airway.

Anesthesia Protocol

In the operating room, peripheral venous access was obtained in the arm contralateral to the neuromuscular monitoring. Patient monitoring consisted of cardioscopy, pulse oximetry, capnometry, capnography, esophageal and peripheral temperature, non-invasive blood pressure (NIBP) in the lower extremity, and bispectral index (BIS®, *Bispectral Index* ^®^ - BIS; *Aspect Medical Systems Inc*., Newton, MA, USA). All patients were warmed up with a thermal sheet over their lower extremities (*Bair Hugger*, *USA*) and core body temperature kept above 95 °F.

After pre-oxygenation (6 l.min^-1^) for 3 minutes under face mask, total venous anesthesia was performed with continuous venous propofol by Agilia®TIVA infusion pump (Fresenius-Kabi, Louviers,France) target-controlled from 2 to 4 ng. mL^-1^, Marsh model, with target guided by the bispectral index, and remifentanil in continuous infusion by Agilia®TIVA infusion pump (Fresenius-Kabi, Louviers, France) at 0.1 to 0.3 ugkg^-1^.min^-1^. Rocuronium 0.6 mg.kg^-1^ intravenously in rapid bolus near the venous access within 5s was injected after the stabilization period. When the TOF was at 0, tracheal intubation was performed and mechanical ventilation started.

Before the end of surgery, nalbufin 0.1 mg.kg^-1^ and ketorolac 30 mg intravenously in bolus were administered for postoperative analgesia. For antiemesis, dexamethasone 4 mg.kg^-1^ and ondansetron 8 mg.kg^-1^ intravenously. The reversal of NB was spontaneous.

With the end of surgery, patients were extubated with a TOF value greater than 0.9 and then referred to the recovery room where they were kept under observation.

Neuromuscular Monitoring

Neuromuscular monitoring was performed by acceleromyograph with the *TOF-Watch SX® monitor* (Organon Ireland Ltd, Dublin, Ireland). TOF sequence was the stimulation pattern used, comparing the last muscle contraction of the adductor pollicis with the first, making a relation of the fourth over the first stimulus (T4/T1). The size of the muscle strength at the first stimulus (T1) was also measured [14].

After skin cleansing, electrodes were placed on the ulnar nerve region near the wrist for stimulation of the adductor pollicis muscle. Preload was used with the use of a hand adapter (Hand Adapter, Organon, Netherlands), which separates the thumb from the index finger and ensures the correct position of the transducer, and immobilization of the hand leaving the thumb free. A temperature sensor was placed over the hypothenar region of the monitored hand. The arm was kept in the same position during the procedure.

A personal computer, by means of an interface, recorded in the TOF program the effect of electrical stimulation of 2 Hz for 1.5 s repeated every 15 s through TOF stimulation: T1, T2, T3, T4.

After induction of general anesthesia and hypnosis assured with the bispectral index below 60, ulnar nerve stimulation with the TOF was initiated for 1 minute. In the control group, calibration was triggered to obtain supramaximal stimulation (electric stimulus applied automatically by the equipment over 15 to 20% for a maximal response) [11] by activating the CAL 2 function in the device for obtaining 100% of T1, guaranteeing that all the muscular fibers were activated. It was then waited for signal stabilization, which consists of a maximum variation of 5% in T1 level maintained for at least 2 minutes. When variance was higher, subsequent calibrations were performed until the stability of the signal was reached. In the tetanic group, before the onset of TOF stimulation, a tetanic stimulus of 50Hz for 5s was applied and then the sequence was initiated as in the control group, with a supramaximal square wave stimulus with 200ms duration, 2Hz frequency and 15s interval between successive TOF stimulation (Figure 1).

Standardization

The numbers at the TOF baseline obtainment after calibration often lie above 100%, so a TOF ratio of 0.9 when evaluating NB reversal does not always translate to blockade recovery. It is then recommended to normalize the TOF value, which consists of relating the values obtained in NB recovery in the TOF to the initial baseline value (first TOF value after calibration) [15]. All NB recovery values described in this study were normalized, according to the good clinical practice recommendation for research on neuromuscular blockers [11].

Figure 1. Neuromuscular Function Monitoring

**
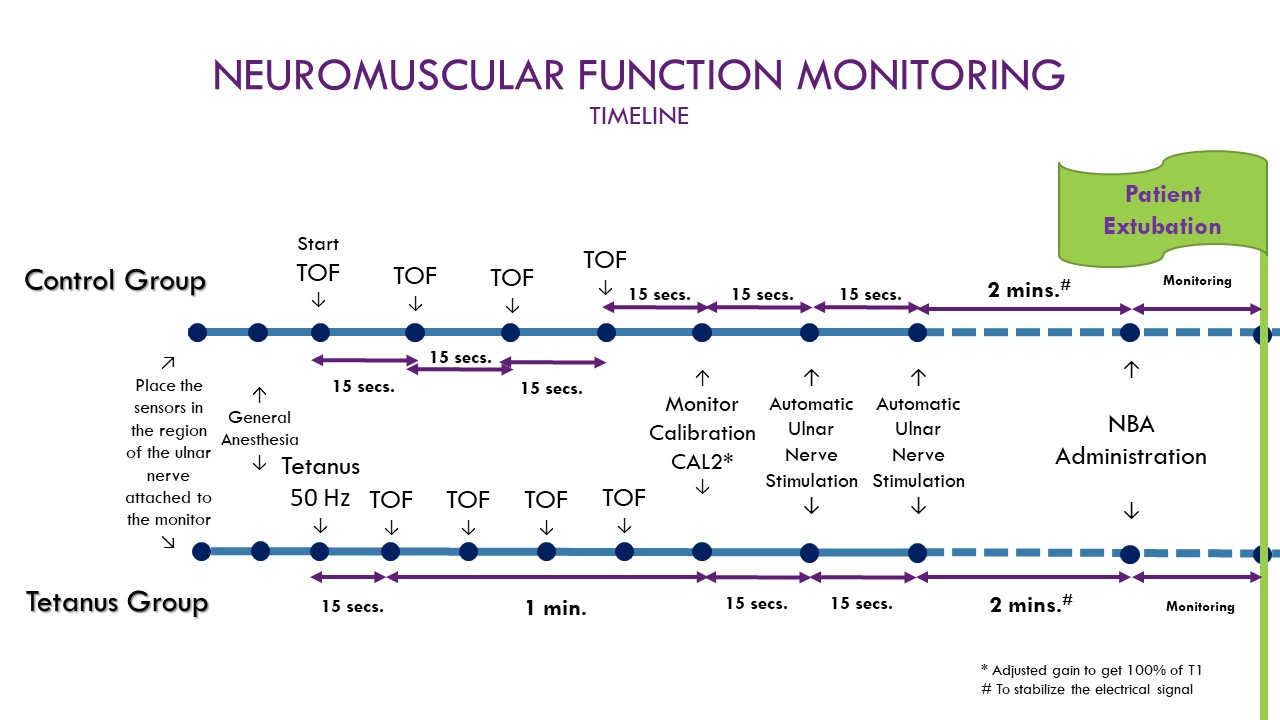
**

Statistical Analysis

Considering a significance level of 5%, a power of 80%, postulated a reduction in the expected stabilization time of 8 min with the intervention, an estimated standard deviation of 10 min, and a 20% estimate for possible losses, based on the study of Carlos et al [16] a total of 30 patients per group was obtained.

Fifty participants completed the study. All results were expressed using absolute and relative frequencies (percentages) for categorical variables or measures of central tendency, and dispersion (mean and standard deviation or median and interquartile range) for numerical variables. In numerical data, the Shapiro-Wilk test was applied to assess whether the variables present normal distribution.

When the continuous variable had a normal distribution, the Student's t-test was used, while the Mann-Whitney test was applied for continuous variables with non-normal distribution. For categorical data, Pearson's chi-square test or Fisher's exact test was used for comparisons of proportions. Pearson's or Spearman's correlation analyses were used to assess the association between numeric and ordinal variables. P values less than 0.05 (p<0.05) were considered statistically significant. Statistical analyses were performed in SPSS 28.0 statistical software (*Statistical Package for Science-Chicago*, IL, 2019).

Outcome Measures

The primary endpoint was the time to obtain stabilization of T1 level, in minutes, when comparing the two groups, with and without tetanic stimulus, for the stabilization of muscle response to electrical stimulation using the TOF-watch SX® acceleromyography monitor. Secondary endpoints were to assess the onset time of NBA action (time measured from rocuronium injection to 95% of T1 height depression) and NB recovery times with or without tetanic stimulus interference. The study settings followed the Stockholm protocol guideline [11].

**Ethics statements**

Respecting the Ethical norms regarding works involving human subjects, this clinical trial was approved by the ethics committees of Hospital Universitário Federal Gaffrée e Guinle (CAAE 03260918.4.0000.5253) and Hospital Universitário Pedro Ernesto (CAAE 03260918.4.3001.5259), Rio de Janeiro, Brazil, registered at clinical trials Brazilian platform REBEC (RBR-35msdt), and informed consents were obtained for each individual participant of this research.

**CRediT author statement**

*CRediT is an initiative that enables authors to share an accurate and detailed description of their diverse contributions to a published work.*

*Example of a CRediT author statement:*

***Zhang San:*** *Conceptualization, Methodology, Software* ***Priya Singh****: Data curation, Writing- Original draft preparation.* ***Wang Wu****: Visualization, Investigation.* ***Jan Jansen:*** *Supervision.* ***Ajay Kumar****: Software, Validation.:* ***Sun Qi:*** *Writing- Reviewing and Editing.*

*Please add a CRediT author statement for your data article here, using the* [*categories listed on this web*](https://www.elsevier.com/authors/journal-authors/policies-and-ethics/credit-author-statement)*page.*

*Angela H S Ribeiro-data curation, writing. NV Figueiredo- editing. PA Germano Filho- supervision. AJMicuci- software,validation. IL Cavalcanti- conceptualization, methodology, reviewing.*

**Acknowledgments**

This research was developed at Hospital Hospital Universitário Pedro Ernesto (HUPE-UERJ) and Hospital Universitário Gafrée e Guinle (HUGG-UNIRIO), Rio de Janeiro-RJ, Brazil. We gratefully acknowledge the support of the Universidade Federal Fluminense, Rio de Janeiro, Brazil; Department of Surgery, Hospital Universitário Pedro Ernesto, UERJ, Rio de Janeiro, Brazil, and Hospital Universitário Gafrée e Guinle, Rio de Janeiro, Brazil.

This research did not receive any specific grant from funding agencies in the public, commercial, or not-for-profit sectors.

**Declaration of interests**

The authors declare that they have no known competing financial interests or personal relationships that could have appeared to influence the work reported in this paper.

**References**

[1] A.L. Plummer-Roberts, C. Trost, S. Collins, I. Hewer, Residual neuromuscular blockade, *AANA Journal.* 84 (2016) 57-65. https://www.aana.com/docs/default-source/aana-journal-web-documents-1/jcourse6-0216-pp57-65.pdf?sfvrsn=1bd448b1_6. (accessed 14 December 2022)

[2] S.R. Thilen, B.E. Hansen, R. Ramaiah, C.D. Kent, M.M. Treggiari, S.M. Bhananker, Intraoperative neuromuscular monitoring site and residual paralysis, *Anesthesiol*. 117 (2012) 964-972. https://doi.org/10.1097/ALN.0b013e31826f8fdd.

[3] G.S. Murphy, S.J. Brull, Residual neuromuscular block: lessons unlearned. Part I: definitions, incidence, and adverse physiologic effects of residual neuromuscular block, *Anesth Analg*. 111 (2010) 120-128. https://doi.org/10.1213/ANE.0b013e3181da832d.

[4] A. Butterly, E.A. Bitter, E. George, W.S. Sandberg, M. Eikermann, U. Schmidt, Postoperative residual curarization from intermediate-acting neuromuscular blocking agents delays recovery room discharge, *Br J Anaesth.* 105 (2010) 304-309. https://doi.org/10.1093/bja/aeq157.

[5] G.S. Murphy, J.W. Szokol, J.H. Marymont, S.B. Greenberg, M.J. Avram, J.S. Vender, M. Nisman, Intraoperative acceleromyographic monitoring reduces the risk of residual neuromuscular blockade and adverse respiratory events in the postanesthesia care unit. *Anesthesiol*. 109 (2008) 389-398. https://doi.org/10.1097/ALN.0b013e318182af3b.

[6] M. Grosse-Sundrup, J.P. Henneman, W.S. Sandberg, B.T. Baterman, J.V. Uribe, N.T. Nguyen, J.M. Ehrenfeld, E.A. Martinez, T. Kurth, M. Eikermann, 2012. Intermediate acting non-depolarizing neuromuscular blocking agents and risk of postoperative complications: prospective propensity score matched cohort study, *BMJ*. 345, e6329. https://doi.org/10.1136/bmj.e6329.

[7] J. Viby-Mogensen, B.C. Jorgensen, H. Ording, Residual curarization in the recovery room, *Anesthesiol*. 50 (1979) 539-541. https://doi.org/10.1097/00000542-197906000-00014.

[8] J.M. Hunter, Reversal of residual neuromuscular block: complications associated with perioperative management of muscle relaxation, *Br J Anaesth*. 119 (2017) i53-i62. https://doi.org/10.1093/bja/aex318.

[9] T. Fuchs-Buder, R. Nemes, D. Schmartz, Residual neuromuscular blockade: management and impacto in postoperative pulmonar outcome, *Curr Opin Anaesthesiol*. 29 (2016) 662-667. https://doi.org/10.1097/ACO.0000000000000395.

[10] A.W. Gelb, W.W Morriss, W. Johnson, A.F. Merry, A. Abayadeera, N. Belîi, S.J. Brull, A. Chibana, F. Evans, C. Goddia, C. Haylock-Loor, F. Khan, S. Leal, N. Lin, R. Merchant, M.W. Newton, J.S. Rowles, A. Sanusi, I. Wilson, A.V. Berumen, World heath Organization –World Federation of Societies of Anaesthesiologists (WHO-WFSA) International Standards for a Safe Practice of Anesthesia, *Anesth Analg*. 126 (2018) 2047-2055. https://doi.org/10.1213/ANE.0000000000002927.

[11] T. Fuchs Buder, L. Claudius, L. Skovgaard, L.I. Erikson, R.K. Mirakhur, J. Viby-Mogensen, Good Clinic Research Practice in pharmacodynamic studies of neuromuscular blocking agents II: the Stockholm Revision, *Acta Anaesthesiol Scand*. 51 (2007) 789-808. https://doi.org/10.1111/j.1399-6576.2007.01352.x.

[12] G.C. Lee, S. Iyengar, J. Szenohradsky, J. E. Caldwell, P.M. Wright, R. Brown, M. Lau, A. Luks, D.M. Fisher, Improving the design of muscle relaxant studies: stabilization period and tetanic recruitment, *Anesthesiol*. 86 (1997) 48-54. https://doi.org/10.1097/00000542-199701000-00008

[13] L.A. Lee, V. Athanassoglou, J.J. Pandit, Neuromuscular blockade in the elderly patient, *J Pain Res*. 9 (2016) 437-444. https://doi.org/10.2147/JPR.S85183

[14] S.J. Brull, A.F. Kopman, Current Status of Neuromuscular Reversal and Monitoring: Challenges and Opportunities, *Anesthesiol*. 126 (2017) 173-190. https://doi.org/10.1097/ALN.0000000000001409.

[15] T. Suzuki, N. Fukano, O. Kitajima, S. Saeki, S. Ogawa, Normalization of acceleromyographic train-of-four ratio by baseline value for detecting residual muscular block, *Br J Anaesth*. 96 (2006) 44-47. https://doi.org/10.1093/bja/aei273.

[16] R.V. Carlos, H.D. de Boer, M.L. Torres, M.J. Carmona, The effect of prior tetanic stimulation on train-of-four monitoring in paediatric patients: a randomized open-label controlled trial, *Eur J Anaesthesiol*. 34 (2017) 163-168. https://doi.org/10.1097/EJA.0000000000000558
