# Supplementary material for: Effect of tetanic stimulation prior to train-of-four monitoring on the time to muscle response stabilization with neuromuscular blockade with rocuronium in patients aged 60 to 80 years: A prospective randomized controlled trial
Source: PLoS One. 2024 Jun 14;19(6):e0303161. doi: 10.1371/journal.pone.0303161 (PMC11178177; doi:10.1371/journal.pone.0303161)
Supplement: Attachment — Submitted filename: PONE-D-23-40644_Response to Reviewers.docx [file pone.0303161.s004.docx]

Lalit Gupta
Academic Editor
PLOS ONE

RE:  PONE-D-23-40644, entitled "Effect of tetanic stimulation prior to train-of-four monitoring on the time to muscle response stabilization in patients aged 60 to 80 years: A prospective randomized controlled trial"

Dear Dr. Lalit Gupta

Thank you very much for your letter dated January 10, 2024, and for the careful review of our manuscript, which we have amended following the reviewers’ suggestions. A marked-up copy of the revised manuscript (with track changes) as well as an unmarked copy of the manuscript (without track changes) have been uploaded to the submission system. Also, please find below an itemized point-by-point response to the reviewers’ comments.

We aimed in this study at the hypothesis that tetanic stimulation would shorten the stabilization time in neuromuscular blockade monitoring in patients aged 60 to 80 years and interfere on the onset time of rocuronium and recovery times, but that was not proven in our study.

We look forward to hearing from you about the status of our manuscript, which we hope is now acceptable for publication in PLOS ONE. Please feel free to contact me if you require any additional information.

Sincerely,

Angela Henrique S. Ribeiro

angelahenriqueanest@gmail.com

­------------------------------------------------------------

*If you would like to make changes to your financial disclosure, please include your updated statement in your cover letter. Guidelines for resubmitting your figure files are available below the reviewer comments at the end of this letter.*

**Response**: No changes were made to our financial disclosure.

*If applicable, we recommend that you deposit your laboratory protocols in* [*protocols.io*](http://protocols.io/) *to enhance the reproducibility of your results. Protocols.io assigns your protocol its own identifier (DOI) so that it can be cited independently in the future.*

**Response:** We thank you for introducing us to protocols.io. However, our data are deposited in Mendeley Database Repository and the original protocol was kept unchanged from the beginning. “Ribeiro AHS, Germano-Filho PA, Cavalcanti I, Figueiredo NV (2022). Effect of tetanic stimulation prior to train-of-four sequence on stabilization time of muscle response in patients aged 60-80. Mendeley Data, V1, doi:10.17632/fyhpfpk5cm.1”

_______________________________________________________________

*Journal Requirements:*

*When submitting your revision, we need you to address these additional requirements.*

*1. Please ensure that your manuscript meets PLOS ONE's style requirements, including those for file naming. The PLOS ONE style templates can be found at* 
[*https://journals.plos.org/plosone/s/file?id=wjVg/PLOSOne_formatting_sample_main_body.pdf*](https://journals.plos.org/plosone/s/file?id=wjVg/PLOSOne_formatting_sample_main_body.pdf) *and* 
[*https://journals.plos.org/plosone/s/file?id=ba62/PLOSOne_formatting_sample_title_authors_affiliations.pdf*](https://journals.plos.org/plosone/s/file?id=ba62/PLOSOne_formatting_sample_title_authors_affiliations.pdf)

*Additional Editor Comments:

The study presented is a multicenter, prospective, randomized, single-blind, controlled trial with a 2-arm parallel group design, aiming to assess the time to stabilization of muscle response with tetanic stimulation in patients aged 60 to 80 years. The authors concluded that tetanic stimulation did not impact the time to T1 twitch stabilization in this age group. However, the manuscript has several deficiencies according to the CONSORT checklist. Specific issues include the lack of information on protocol changes, incomplete reporting in the abstract, insufficient p-value precision, the suggestion of using a Linear Mixed Effects Model for data structure, unclear definition of "stabilization," missing details on randomization, inadequate sample size justification, unclear statistical models in tables, and the need for dose-response modeling for certain data. Additionally, some statements and sections require clarification and rephrasing. Overall, the manuscript requires thorough revisions to address these issues and improve its quality.*

**Response**: Thank you for the comments, we altered the article and made the corrections according to your guidance.

Comments to the Author

**1. Is the manuscript technically sound, and do the data support the conclusions?**

*The manuscript must describe a technically sound piece of scientific research with data that supports the conclusions. Experiments must have been conducted rigorously, with appropriate controls, replication, and sample sizes. The conclusions must be drawn appropriately based on the data presented.*

*Reviewer #1: Yes*

*Reviewer #2: Partly*

**2. Has the statistical analysis been performed appropriately and rigorously?**

*Reviewer #1: Yes*

*Reviewer #2: I Don't Know*

**3. Have the authors made all data underlying the findings in their manuscript fully available?**

*The* [*PLOS Data policy*](http://www.plosone.org/static/policies.action#sharing) *requires authors to make all data underlying the findings described in their manuscript fully available without restriction, with rare exception (please refer to the Data Availability Statement in the manuscript PDF file). The data should be provided as part of the manuscript or its supporting information, or deposited to a public repository. For example, in addition to summary statistics, the data points behind means, medians and variance measures should be available. If there are restrictions on publicly sharing data—e.g. participant privacy or use of data from a third party—those must be specified.*

*Reviewer #1: Yes*

*Reviewer #2: No*

**Response**: We thank you for your questions and suggestions. All data underlying the findings described in the manuscript are fully available without restriction. The data collected for the research were deposited in a public repository, Mendeley Database Repository: “Ribeiro AHS, Germano-Filho PA, Cavalcanti I, Figueiredo NV (2022). Effect of tetanic stimulation prior to train-of-four sequence on stabilization time of muscle response in patients aged 60-80”. *Mendeley Data*, V1, doi:10.17632/fyhpfpk5cm.1.”

The data points behind means, medians and variance measures are deposited in this public repository. There are no restrictions on publicly sharing data.

**4. Is the manuscript presented in an intelligible fashion and written in standard English?**

*PLOS ONE does not copyedit accepted manuscripts, so the language in submitted articles must be clear, correct, and unambiguous. Any typographical or grammatical errors should be corrected at revision, so please note any specific errors here.*

*Reviewer #1: Yes*

*Reviewer #2: No*

**Response**: We are not native English speakers, so we used the services of a specialized company, American Journal Experts (AJE) – Springer Nature, registered SKLF32XNL, Sep 2023. We have uploaded AJE’s Editorial Certificate to the submission system as Supplementary Information.

5. Review Comments to the Author

Please use the space provided to explain your answers to the questions above. You may also include additional comments for the author, including concerns about dual publication, research ethics, or publication ethics. (Please upload your review as an attachment if it exceeds 20,000 characters)

Reviewer #1: This study provides useful details for neuromuscular monitoring that has not been investigated yet.
Two comments: rocuronium should be mentioned in the title.

**Response**: Thank you for your considerations and suggestions. We included rocuronium in the title as suggested (page 1, lines 1-3).

The second does not influence the results but as there is considerable difference between plasma or effect-site in TIVA, in page 10, line 151 it should be mentioned whether you used Marsh plasma-site (I suppose) or effect-site concentrations.

Response: We used Marsh pharmacokinetic model in this research. Included in the manuscript (page 10, line 161).

Reviewer #2: The authors reported the results of multicenter, prospective, randomized, single-blind, controlled trial with a 2 arm parallel group design to determine the time to stabilization of the muscle response with tetanic stimulation compared to no stimulation in patients aged 60 to 80 year. They concluded that the use of tetanic stimulation to shorten the time to T1 height stabilization in neuromuscular monitoring did not alter the stabilization time of the T1 twitch in patients between 60 and 80 years old.

In general, the current presentation has deficits and needs a sound revisions.

general: Please comment on each point of the CONSORT checklist in the manuscript. E.g. if no changes to the protocol are made, please state this explicitly.

Response: Thank you for your considerations and suggestions. Each point of the CONSORT checklist was revised as requested, and the suggested revisions were made to the manuscript. No changes to the anesthesia protocol or methods were made (page 7, lines 92-93). We just added another site center due to difficulty recruiting during the pandemic.

L27-32: Please give the information required in the CONSORT abstract form here, e.g. setting, primary endpoint variable, randomization technique, etc.

**Response**: The information required in the CONSORT abstract form was provided in the revised manuscript (page 3, lines 26-36).

L34ff: Please give p-values with at least 3 decimal digits.

**Response**: P-values with at least 3 decimal digits were provided throughout the revised manuscript.

L36-39: data seem to follow a "repeated" measures type of structure, which should be reflected in the analysis by a Linear Mixed Effects Model.

**Response**: The measurements were not repeated but rather consecutive. The neuromuscular blockade recovery times measured are not comparable. TOF 40 is muscle strength recovery of 40% of baseline, TOF 50 is muscle strength recovery of 50% of baseline, TOF 60 is muscle strength recovery of 60% of baseline, TOF 70 is muscle strength recovery of 70% of baseline, TOF 80 is muscle strength recovery of 80% of baseline, TOF 90 is muscle strength recovery of 90% of baseline, up to total muscle strength recovery of 100% of TOF.

L76-80: First as biostatistician I am not sure about the objective definition of stabilization, which is necessary to determine the time. In particular, it is unclear, what is meant by " stabilization", whether " stabilization" was observed in every patient, whether the "degree of stabilization" is the same with every patient. Could you please provide more information.

**Response**: According to the Stockholm Revision (ref 7), muscle response stabilization is defined as a variation of less than 5% in T1 height (first twitch) in response to the electrical stimulus applied to the corresponding nerve. The stabilization time used in this study was defined as the period of time between the end of calibration (adjusted twitch height to 100%) and achievement of a muscle response variation (T1-first twitch) of less than 5% for at least 2 minutes (page 6, lines 69-70). The same stabilization concept was used for all patients. The time to reach stabilization is individual, so each patient will have their own time to stabilize the muscle response to the electrical stimulus. The characteristics of the electrical stimulus that all patients received were standardized; what varied was the time for the muscle response to stabilize, as this is an individual organic response for each patient. The neuromuscular blockade is monitored by applying a small electric shock in a sequence of four stimuli, leading to four muscle contractions, known as train-of-four (TOF). The electric shock to the peripheral nerve sends a signal for the corresponding muscle to contract. In this study, we used stimulation of the ulnar nerve to contract the adductor pollicis (page 10, lines 149-151; page 11, lines 164-165).

­­­­­­­­­­­

*Second, the specific hypothesis to be proven is not given.*

**Response**: The hypothesis was added to the manuscript (page 6, lines 77-78). Our hypothesis was that the use of tetanic stimulus prior to the sequence of four stimuli would decrease the stabilization time of muscular response and alter the onset and recovery times of the neuromuscular blockade.

L98-101: As enrollment starts at different times in the two centers, it is likely to assume a time trend in the data. So please provide at least a figure of the treatment effect by center to make clear that the treatment effect is consistent across centers.

**Response**: Enrollment started at different times in the two centers, but there was no time trend in the data. The measurements were independent and individual, starting and ending during the same anesthetic-surgical procedure. There was no time effect on the data.

L120-125: Please give details of the randomization. Missing details cover the randomization algorithm (e.g. permuted block randomization with block size 4?). It is unclear, how the random allocation is implemented, e.g. by the sealed opaque envelops (?), and who provided the random lots, who assigned patients to make concealment of randomization clear. The technique to conceal and protect the information about the allocation from the patients (blinding) need to be described.

Response: The randomization was made with 60 random numbers generated in the computer program WinPepi (Program for Epidemiologists for Windows) for the two groups. The study participants were not aware of the allocated group, as they were anesthetized, unconscious, and therefore blinded to the allocation. We obeyed the random allocation sequence generated by the program. The random allocation was implemented by sealed opaque envelopes, which were opened by a single research anesthesiologist after anesthesia induction. Blinding at this point was not possible. The technique to conceal and protect the information about the allocation from the patients was detailed in the revised manuscript, as suggested by the reviewer (page 9, lines 127-135).

L199-203: Sample size justification need further information. The statistical test underlying the sample size justification is missing. Please state the software used for calculation. The result might be to low, as - assuming a two sample t-Test - a sample size of 26 / group is necessary without 20% dropout. Please note, that a dropout rate of 20% is rather large and may cause the validity. Please comment on these points in the limitation section, as the study might appear as underpowered.

Response: Student’s t-test was used to calculate the sample size (page 13, line 211). To use this test, it was necessary to determine the expected effect size (variable: electrical signal stabilization time), which is the ratio of the difference between the means of the two groups to the common standard deviation of the difference. By setting an effect size of 0.8 standard deviations, we estimated this effect for the underlying test. The means for the intervention and control groups were 2.5 (SD, 1.0) and 5.0 (SD, 5.2), respectively, in the pilot study with 10 patients, 5 in each group. Selection bias should be avoided, but a 20% loss is usually acceptable in studies, as reported in the following article: “Schulz KF, Grimes DA. Sample size slippages in randomized trials: exclusions and the lost and wayward. *Lancet* 2002; 359(9308): 781-5.”

We included the dropout rate in the limitations section (page 22, line 344-345).

L204ff: Please make the section specific to the content so to avoid general description and state which method is used in which situation. Give the statistical analysis of the primary endpoint variable. Give the analysis population and state how to deal with missing values to avoid attrition bias. Use the ITT population.
Pretest on normal distribution might be appropriate for explorative analysis, i.e. ancillary analysis.

Response: We stated which method is used in each situation and gave the statistical analysis of the primary endpoint variable in the revised manuscript. The data were normally distributed according to the Shapiro-Wilk test. Student’s t test was used to compare normally distributed continuous variables (age, BMI, and creatinine clearance) between the intervention and control groups. For categorical variables, Pearson’s chi-square test or Fisher's exact test was used to compare proportions (sex and ASA class) between the intervention and control groups. Due to the different outcomes studied, multivariate analysis of variance (MANOVA) with Bonferroni adjustment was applied in the comparison between groups to control for type I error (time to stabilization, onset time, initial and final T1 height, clinical duration, and neuromuscular blockade recovery variables) (pages 13-14, lines 216-223). The primary endpoint was not measured in the excluded patients. The result of the intervention is immediate, not prolonged, which is why we did not consider the ITT population (page 14, lines 232-234).

­­­­­­­­­­­­­­­­­­­­

L255: The basis should be 30 patients in each group according to flow chart. Statistical test for baseline patient characteristics are not meaningful in randomized clinical trials, delete last column of table 1.

**Response**: Participants who were excluded after allocation were not analyzed for the primary outcome (page 14, lines 232-234). We have deleted the last column of table 1.

L235: If MANOVA is applied there is more information needed to understand the statistical model. I assume the first row "Time .." just refers to the t-Test (underlying the sample size justification) and thus the p-value is from a t-Test. Please give means and SD for each group followed by a 95% confidence interval for mean difference. How are missing values dealed with. Last row could be deleted.

**Response**: Because we evaluated multiple dependent variables (all times) between the groups, MANOVA is a multivariate analysis that controls for type I error, verifies the group effect by comparing multiple outcomes at the same time for the same factor, adjusting for recovery times that are related to each other. The t-test would compare each time separately, whereas MANOVA does this simultaneously.

The means for the intervention and control groups were 2.5 (SD, 1.0) and 5.0 (SD, 5.2), respectively, in the pilot study with 10 patients, 5 in each group. The “stabilization time” variable was asymmetric, with a 95% CI of 3.1-6.6 for the intervention group and 5.2-8.5 for the control group (page 15, Table 2). There were no missing values (page 14, line 234).

L244: Unclear what % means in row 2 and 3. With % numbers the column entries are difficult to understand. May bet this is an ANCOVA model.
Like in Table 2 MANOVA model not clear, please give the within and between factors, correlation structure and other model parameters.

Response: The % mentioned in the table means that the value of the initial and final T1 height is a percentage by concept, not by mathematical calculation (ref 15: Carlos RV, de Boer HD, Torres MLA, Carmona MJC. The effect of prior tetanic stimulation on train-of-four monitoring in paediatric patients. Eur J Anaesthesiol. 2017;34: 163-168). The only effect to be assessed is between groups, which is why we used MANOVA as detailed in the previous response.

L256: Data for "Time to a normalized TOF value of" seem to be a dose response profile and should be modelled accordingly to establish differences.

Response: Time to a normalized TOF value is not a dose response, but a correction to the participant’s baseline TOF values to increase the accuracy of the data and, therefore, confirm clinical recovery from neuromuscular blockade. According to the Stockholm Revision, the concept of normalization is as follows: “During recovery, the twitch height does not always return to the control value. Therefore, all recovery parameters based on the twitch height (i.e., duration 25%) should be adjusted to the final T1 value (normalization). If, for instance, the final T1 is 80%, a recorded T1 value of 20% should be adjusted to 25% (0.20/0.80).” (Ref 7: Fuchs-Buder T, Claudius C, Skovgaard LT, Eriksson LI, Mirakhur RK, Viby-Mogensen J. Good clinical research practice in pharmacodynamic studies of neuromuscular blocking agents II: the Stockholm revision. Acta Anaesthesiol Scand. 2007;51: 789-808).

L338: Statement is not clear and need rephrasing.

Response: We rephrased it in the manuscript: the sample size was only calculated for the primary outcome (page 22, lines 344-345).
