# Supplementary material for: Effect of tetanic stimulation prior to train-of-four monitoring on the time to muscle response stabilization with neuromuscular blockade with rocuronium in patients aged 60 to 80 years: A prospective randomized controlled trial
Source: PLoS One. 2024 Jun 14;19(6):e0303161. doi: 10.1371/journal.pone.0303161 (PMC11178177; doi:10.1371/journal.pone.0303161)
Supplement: Attachment — Submitted filename: PONE-D-23-40644R1_Response to Reviewers.docx [file pone.0303161.s005.docx]

Lalit Gupta
Academic Editor
PLOS ONE

RE:  PONE-D-23-40644R1, entitled "Effect of tetanic stimulation prior to train-of-four monitoring on the time to muscle response stabilization with neuromuscular blockade with rocuronium in patients aged 60 to 80 years: A prospective randomized controlled trial"

Dear Dr. Lalit Gupta

Thank you for your email dated March 8, 2024, and once again for the careful review of our manuscript, which we have amended following the reviewer’s suggestions. A marked-up copy of the revised manuscript (with track changes) as well as an unmarked copy of the manuscript (without track changes) have been uploaded to the submission system. Also, please find below an itemized point-by-point response to the reviewers’ comments.

We look forward to hearing from you about the status of our manuscript, which we hope is now acceptable for publication in PLOS ONE. Please feel free to contact me if you require any additional information.

Sincerely,

Angela Henrique S. Ribeiro

angelahenriqueanest@gmail.com

==============================

Additional Editor Comments:

The study aimed to determine the time to stabilization of muscle response with tetanic stimulation compared to no stimulation in patients aged 60 to 80 years. However, the use of tetanic stimulation did not alter the stabilization time of the T1 twitch in this age group. Several points need clarification, including the definition of “stabilization,” specific hypotheses, and randomization details. Additionally, addressing the time trend across centers would enhance the analysis.

***Response****: Thank you for your considerations. We clarified those points in the text as required. More specifically, the definition of “stabilization” is fully explained in the Stockholm Revision Guidelines for researchers (ref 7 of the manuscript), and our study strictly followed the guidelines. We clarified in the revised manuscript the definition of stabilization, as defined by the guidelines (page 6, lines 69-70). Also, the specific hypotheses were altered as requested by the reviewer (page 6, lines 77-78). Randomization details were added in the previous round as requested by the reviewers, and complemented in this round (page 9, lines 127-136). Regarding the issue about time trend across centers, raised in the previous round of review, we explained that, although enrollment started at different times in the two centers, there was no time trend in the data because the measurements were made independently and individually, starting and ending in the same anesthetic-surgical procedure. Therefore, there was no time effect on the data.*


[Note: HTML markup is below. Please do not edit.]

Reviewers' comments:

Reviewer's Responses to Questions

**Comments to the Author**

1. If the authors have adequately addressed your comments raised in a previous round of review and you feel that this manuscript is now acceptable for publication, you may indicate that here to bypass the “Comments to the Author” section, enter your conflict of interest statement in the “Confidential to Editor” section, and submit your "Accept" recommendation.

Reviewer #1: (No Response)

Reviewer #2: (No Response)

2. Is the manuscript technically sound, and do the data support the conclusions?

The manuscript must describe a technically sound piece of scientific research with data that supports the conclusions. Experiments must have been conducted rigorously, with appropriate controls, replication, and sample sizes. The conclusions must be drawn appropriately based on the data presented.

Reviewer #1: (No Response)

Reviewer #2: Partly

3. Has the statistical analysis been performed appropriately and rigorously?

Reviewer #1: (No Response)

Reviewer #2: No

4. Have the authors made all data underlying the findings in their manuscript fully available?

The [PLOS Data policy](http://www.plosone.org/static/policies.action#sharing) requires authors to make all data underlying the findings described in their manuscript fully available without restriction, with rare exception (please refer to the Data Availability Statement in the manuscript PDF file). The data should be provided as part of the manuscript or its supporting information, or deposited to a public repository. For example, in addition to summary statistics, the data points behind means, medians and variance measures should be available. If there are restrictions on publicly sharing data—e.g. participant privacy or use of data from a third party—those must be specified.

Reviewer #1: (No Response)

Reviewer #2: No

5. Is the manuscript presented in an intelligible fashion and written in standard English?

PLOS ONE does not copyedit accepted manuscripts, so the language in submitted articles must be clear, correct, and unambiguous. Any typographical or grammatical errors should be corrected at revision, so please note any specific errors here.

Reviewer #1: (No Response)

Reviewer #2: Yes

6. Review Comments to the Author

Please use the space provided to explain your answers to the questions above. You may also include additional comments for the author, including concerns about dual publication, research ethics, or publication ethics. (Please upload your review as an attachment if it exceeds 20,000 characters)

Reviewer #1: (No Response)

Reviewer #2: L77: The hypotheses refers to three objectives = criteria or primary endpoint variables, which is not in line with the methodological approach. Thus only one variable should be mentioned " We hypothesized that the tetanic stimulation could decrease stabilization time." .

***Response****: Thank you for your considerations. We changed the text as suggested, with just one variable mentioned (page 6, lines 77-78).*


L211: Sample size justification is not correct. The provided sample size calculation relies on the z-Test rather than on the t-Test. Conditioned on the assumed effect size, the dropout rate needs to be added, which results in a total sample size of 62.5 in case of the z-test and 65 in case of the t-test. Thus, in the limitation section this needs to be stated resulting in the conclusion, that the study is underpowered.

***Response****: Thank you for your considerations. Due to the paucity of data in the literature, the sample size was calculated based on a pilot study with 10 patients. Considering a significance level of 5% and a power of 80% to detect a reduction in the time to stabilization of the expected T1 height within 8 minutes after the tetanic stimulation, an estimated standard deviation of 10 minutes (minimum effect size of 0.8 standard deviations) and an estimated 20% of potential losses, a sample size of 30 patients per group was needed (page 13, lines 211-215). If we had achieved an effect size of 0.8 standard deviations, we would have found statistical significance, but the effect size found in the study was 0.5. Therefore, the “n” was not sufficient because the effect found was smaller than that initially obtained in the pilot study and not because of an error in the sample size calculation. As requested, we included this explanation in the limitation section (page 22, lines 344-348).*

L204ff: In the statistical method section delete the test on normal distribution, as the test is not meaningfull for analysis of the primary endpoint.

***Response****: Thank you for your considerations. We excluded from the statistical method section the test on normal distribution as required (page 14, line 220).*


The analysis population is the Per Protocol population.

Please state in the limitation section, that the analysis population, as per protocol, results in anticonservatve test decision.

***Response****: Thank you for your consideration. As required, we included in the limitation section that the analysis population, as per protocol, resulted in anticonservative test decision (page 22, line 346).*

7. PLOS authors have the option to publish the peer review history of their article ([what does this mean?](https://journals.plos.org/plosone/s/editorial-and-peer-review-process#loc-peer-review-history)). If published, this will include your full peer review and any attached files.


If you choose “no”, your identity will remain anonymous but your review may still be made public.


**Do you want your identity to be public for this peer review?** For information about this choice, including consent withdrawal, please see our [Privacy Policy](https://www.plos.org/privacy-policy).

Reviewer #1: No

Reviewer #2: No

[NOTE: If reviewer comments were submitted as an attachment file, they will be attached to this email and accessible via the submission site. Please log into your account, locate the manuscript record, and check for the action link "View Attachments". If this link does not appear, there are no attachment files.]

While revising your submission, please upload your figure files to the Preflight Analysis and Conversion Engine (PACE) digital diagnostic tool, <https://pacev2.apexcovantage.com/>. PACE helps ensure that figures meet PLOS requirements. To use PACE, you must first register as a user. Registration is free. Then, login and navigate to the UPLOAD tab, where you will find detailed instructions on how to use the tool. If you encounter any issues or have any questions when using PACE, please email PLOS at [figures@plos.org](mailto:figures@plos.org). Please note that Supporting Information files do not need this step.
